# Mimicking clinical trials with synthetic acute myeloid leukemia patients using generative artificial intelligence

**DOI:** 10.1038/s41746-024-01076-x

**Published:** 2024-03-20

**Authors:** Jan-Niklas Eckardt, Waldemar Hahn, Christoph Röllig, Sebastian Stasik, Uwe Platzbecker, Carsten Müller-Tidow, Hubert Serve, Claudia D. Baldus, Christoph Schliemann, Kerstin Schäfer-Eckart, Maher Hanoun, Martin Kaufmann, Andreas Burchert, Christian Thiede, Johannes Schetelig, Martin Sedlmayr, Martin Bornhäuser, Markus Wolfien, Jan Moritz Middeke

**Affiliations:** 1 Department of Internal Medicine I, University Hospital Carl Gustav Carus, Technical University Dresden, Dresden, Germany; 2 Else Kröner Fresenius Center for Digital Health, Technical University Dresden, Dresden, Germany; 3 Center for Scalable Data Analytics and Artificial Intelligence (ScaDS.AI) Dresden/Leipzig, Leipzig, Germany; 4 Institute for Medical Informatics and Biometry, Technical University Dresden, Dresden, Germany; 5 Medical Clinic and Policlinic I Hematology and Cell Therapy, University Hospital, Leipzig, Germany; 6 Department of Medicine V, University Hospital Heidelberg, Heidelberg, Germany; 7 Department of Medicine 2, Hematology and Oncology, Goethe University Frankfurt, Frankfurt, Germany; 8 Department of Hematology and Oncology, University Hospital Schleswig Holstein, Kiel, Germany; 9 Department of Medicine A, University Hospital Münster, Münster, Germany; 10 Department of Internal Medicine V, Paracelsus Medizinische Privatuniversität and University Hospital Nürnberg, Nürnberg, Germany; 11 Department of Hematology, University Hospital Essen, Essen, Germany; 12 Department of Hematology, Oncology and Palliative Care, Robert-Bosch-Hospital, Stuttgart, Germany; 13 Department of Hematology, Oncology and Immunology, Philipps-University-Marburg, Marburg, Germany; 14 German Consortium for Translational Cancer Research DKFZ, Heidelberg, Germany; 15 National Center for Tumor Diseases (NCT), Dresden, Germany

**Keywords:** Acute myeloid leukaemia, Clinical trials

## Abstract

Clinical research relies on high-quality patient data, however, obtaining big data sets is costly and access to existing data is often hindered by privacy and regulatory concerns. Synthetic data generation holds the promise of effectively bypassing these boundaries allowing for simplified data accessibility and the prospect of synthetic control cohorts. We employed two different methodologies of generative artificial intelligence – CTAB-GAN+ and normalizing flows (NFlow) – to synthesize patient data derived from 1606 patients with acute myeloid leukemia, a heterogeneous hematological malignancy, that were treated within four multicenter clinical trials. Both generative models accurately captured distributions of demographic, laboratory, molecular and cytogenetic variables, as well as patient outcomes yielding high performance scores regarding fidelity and usability of both synthetic cohorts (*n* = 1606 each). Survival analysis demonstrated close resemblance of survival curves between original and synthetic cohorts. Inter-variable relationships were preserved in univariable outcome analysis enabling explorative analysis in our synthetic data. Additionally, training sample privacy is safeguarded mitigating possible patient re-identification, which we quantified using Hamming distances. We provide not only a proof-of-concept for synthetic data generation in multimodal clinical data for rare diseases, but also full public access to synthetic data sets to foster further research.

## Introduction

In the age of big data, the paucity of publicly available medical data sets is often staggering. Despite extensive data collection efforts, such as The Cancer Genome Atlas^1^, the public availability of comprehensive entity-specific data sets remains largely unsatisfactory. Data sharing is often hindered by concerns of patient privacy, regulatory aspects, and proprietary interests^2^. These factors do not only impede progress in medical research but also establish a gatekeeping mechanism that restricts specific research inquiries to large institutions with access to extensive datasets. Collecting such data sets is a costly and time-consuming effort and especially later-phase clinical trials usually take years to complete and require millions in funding^3,4^. In particular, this is true for rare diseases, such as acute myeloid leukemia (AML), which is a genetically heterogenous and highly aggressive hematological malignancy with so far unsatisfactory patient outcomes despite recent advances in therapy^5^. In addition, the development of targeted therapies for defined subgroups leads to an increased need for control groups^6^. To gain insights into such burdensome malignant entities with unmet medical needs, a crowd-sourcing of data to refine risk stratification efforts and test treatment-related hypothesis is essential. If machine learning methods are to be deployed in such data sets, the size of available diverse training data is paramount for model robustness. Generative models, especially generative adversarial neural networks (GANs)^7^, have exhibited remarkable capabilities in image generation^8^, but can also effectively generate synthetic non-image data. The unique properties of generative artificial intelligence (AI) yield the prospect of synthesizing data based on real patients, which can be distributed at will since, ideally, synthetic data only mimics real patient data alleviating concerns of privacy. In this scenario, the synthetic data itself should preserve the biological characteristics of the disease under investigation to make inferences to real-world applications possible. At the same time, synthetic data should safeguard privacy of the underlying training cohort.

In this study, we employ two state-of-the-art technologies of generative modeling on a large training data set of four pooled multicenter clinical trials including AML patients with comprehensive clinical and genetic information. We investigate how closely the synthetic data resembles the real trial data aligning baseline characteristics and patient outcome. Further, we measure privacy conservation in the synthetic data. Additionally, we provide both final fully synthetic data sets comprising 1606 AML patients each in a publicly accessible repository to foster further research into this devastating disease.

## Results

### Synthetic cohorts generated by CTAB-GAN+ and NFlow score highly in fidelity metrics

We generated equally sized data sets of *n* = 1606 synthetic patients with each generative model to compare patient variables to the original cohort. The fidelity of synthetic data was assessed with three previously proposed performance metrics scaled from 0 (inadequate representation) to 1 (optimal representation). First, the distribution of each individual variable was compared between original and synthetic data again yielding high scores for both models (Regularized Support Coverage^9^ for CTAB-GAN+: 0.95 and NFlow: 0.97). Second, continuous numerical variables were assessed by comparing mean, median, and standard deviation between original and synthetic data per variable (Basic Statistical Measure^9^) showing high scores for both CTAB-GAN+ (0.91) and NFlow (0.92). Third, regarding accurate representations of inter-variable correlations, CTAB-GAN+ and NFlow achieved a Log-Transformed Correlation Score^9^ of 0.75 and 0.74, respectively. An overview of performance metrics is provided in Supplementary Table 1 (usability; survival metrics are reported with survival analysis).

### Synthetic clinical and genetic patient characteristics closely mimic those of real patients

Baseline patient characteristics compared between real and synthetic patients are shown in Table 1. The distribution of patients from different trials between training and test set did not differ significantly (Supplementary Table 2). It has to be noted that given the large sample sizes (three groups with *n* = 1606 each), even small effect sizes yield statistically significant differences. For instance, median age in the original cohort was 56 years, while synthetic patients generated by CTAB-GAN+ had a slightly younger median age of 53 years (*p* = 0.0001), whereas NFlow-generated patients had a slightly older median age of 58 years (*p* = 0.039). Sex distribution did not differ between NFlow and the original cohort, while CTAB-GAN+ generated more males than females (NFLOW: 56.2% vs. 43.8%; original: 52.2% vs. 47.8%; *p* = 0.023). The rates of de novo, secondary, and therapy-associated AML did not differ significantly for CTAB-GAN+ generated patients, while NFlow generated fewer de novo and more therapy-associated AML patients compared to the original cohort. Hemoglobin levels and platelet count did not differ significantly between the original and the synthetic cohorts, while synthetic patients generated by CTAB-GAN+ showed a significantly higher median white blood cell count than the original cohort. Notably, the way outliers were handled regarding continuous variables (age, WBC, PLT, Hb) was different for both models compared to the original data. In the original data set, the number of patients with outliers at the upper end of the spectrum was thinned out as more extreme values were less likely. This behavior was better represented by NFlow than by CTAB-GAN+ (Supplementary Fig. 1). Especially for WBC, CTAB-GAN+ seemed to even out the outliers across the upper distribution range resulting in a statistically significant difference compared to the original cohort (Table 1) whereas outliers for Nflow were more in line with the original cohort. Interestingly, at the same time CTAB-GAN+ completely cuts off outliers roughly below the 600 GPt/l mark for PLT.

**Table 1:** Distribution of baseline characteristics between the original and synthetic cohort

Clinical data	original cohort	CTAB-GAN +	*p*	NFlow	*p*
Number of patients	1606	1606		1606	
Age, median (IQR)	56 (44–65)	53 (42–64)	**0.0001**	58 (47–66)	**0.039**
Sex, *n* (%)			**0.023**		0.672
Female	768 (47.8)	703 (43.8)		781 (48.6)	
Male	838 (52.2)	903 (56.2)		825 (51.4)	
AML status, n (%)					
de novo	1339 (83.4)	1339 (83.4)	1.000	1250 (77.8)	**0.041**
Secondary	195 (12.1)	193 (12.0)	0.914	200 (12.5)	0.554
Therapy-associated	54 (3.4)	57 (3.5)	0.847	83 (5.2)	**0.007**
Extramedullary disease, *n* (%)	224 (13.9)	228 (14.2)	0.409	279 (17.4)	**0.003**
ELN2022, *n* (%)					
Favorable	515 (32.1)	–		–	
Intermediate	449 (28.0)	–		–	
Adverse	624 (38.9)	-		–	
**Laboratory values**					
WBC, median (IQR) in GPt/l	19.5 (4.5–53.4)	27.0 (8.3–69.6)	**<0.0001**	14.4 (5.8–55.3)	0.832
Hb, median (IQR) in mmol/l	5.9 (5.0–8.6)	5.8 (5.0–7.0)	0.949	5.9 (5.2–6.8)	0.988
Plt, median (IQR) in GPt/l	50.0 (27.0–94.0)	49.7 (31.0–93.4)	0.073	48.0 (26.2–94.5)	0.405

Boldface indicates statistical significance (*p* < 0.05). *p*-values are calculated using two-sample comparisons between each of the synthetic cohorts and the baseline cohort for reference. *Hb* hemoglobin, *IQR* interquartile range; *n* number, *Plt* platelet count, *WBC* white blood cell count.

**Fig. 1:**
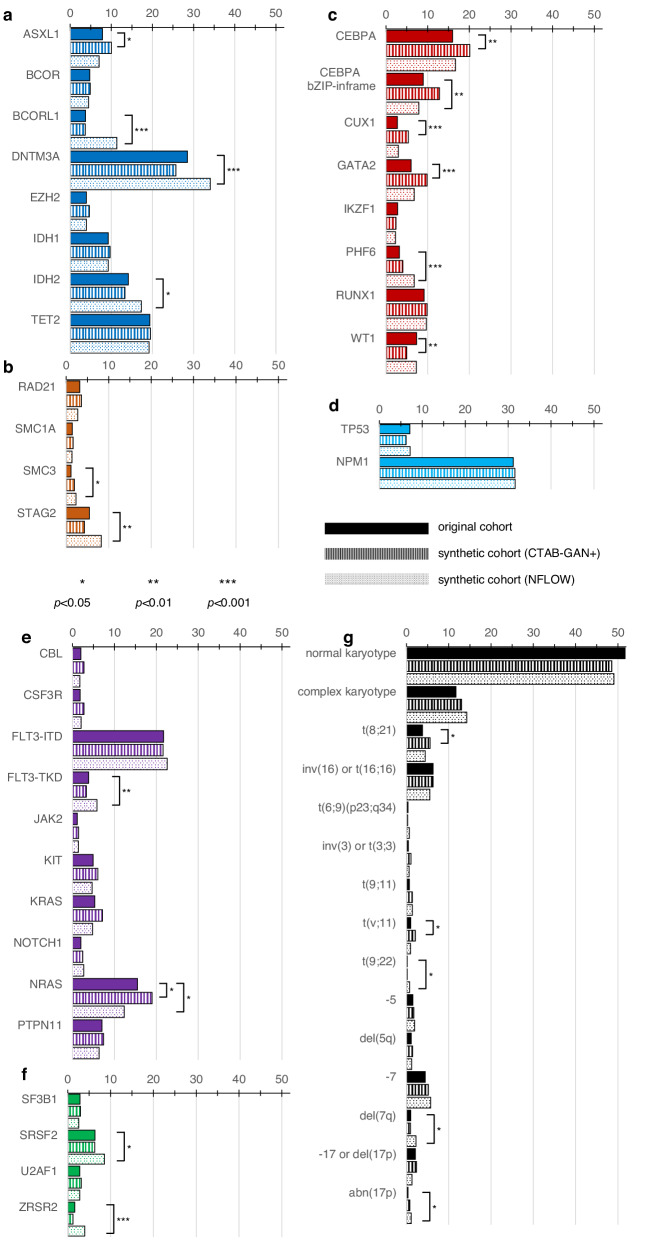
Distribution of molecular and cytogenetic alterations between real and synthetic patients. 50 molecular genetic and cytogenetic alterations were included in generative modeling. Molecular genetics were originally assessed by next-generation sequencing using a targeted myeloid panel including genes that encode for epigenetic regulators (**a**, dark blue), the cohesion complex (**b**, orange), transcription factors (**c**, red), NPM1 and TP53 (**d**, light blue), signaling factors (**e**, purple), and the spliceosome (**f**, green). Cytogenetic aberrations (**g**, black) were selected based on previously demonstrated impact on patient outcomes. Distributions for all variables are denoted as percentages of each respective cohort. Overall, both synthetic cohorts well represented the distribution of alterations in the original cohort with only slight deviations denoted by highly statistically significant (*p* < 0.001) differences in *BCORL1, DNMT3A, PHF6*, and *ZRSR2* for NFlow, as well as *CUX1* and *GATA2* for CTAB-GAN + .

**Fig. 2:**
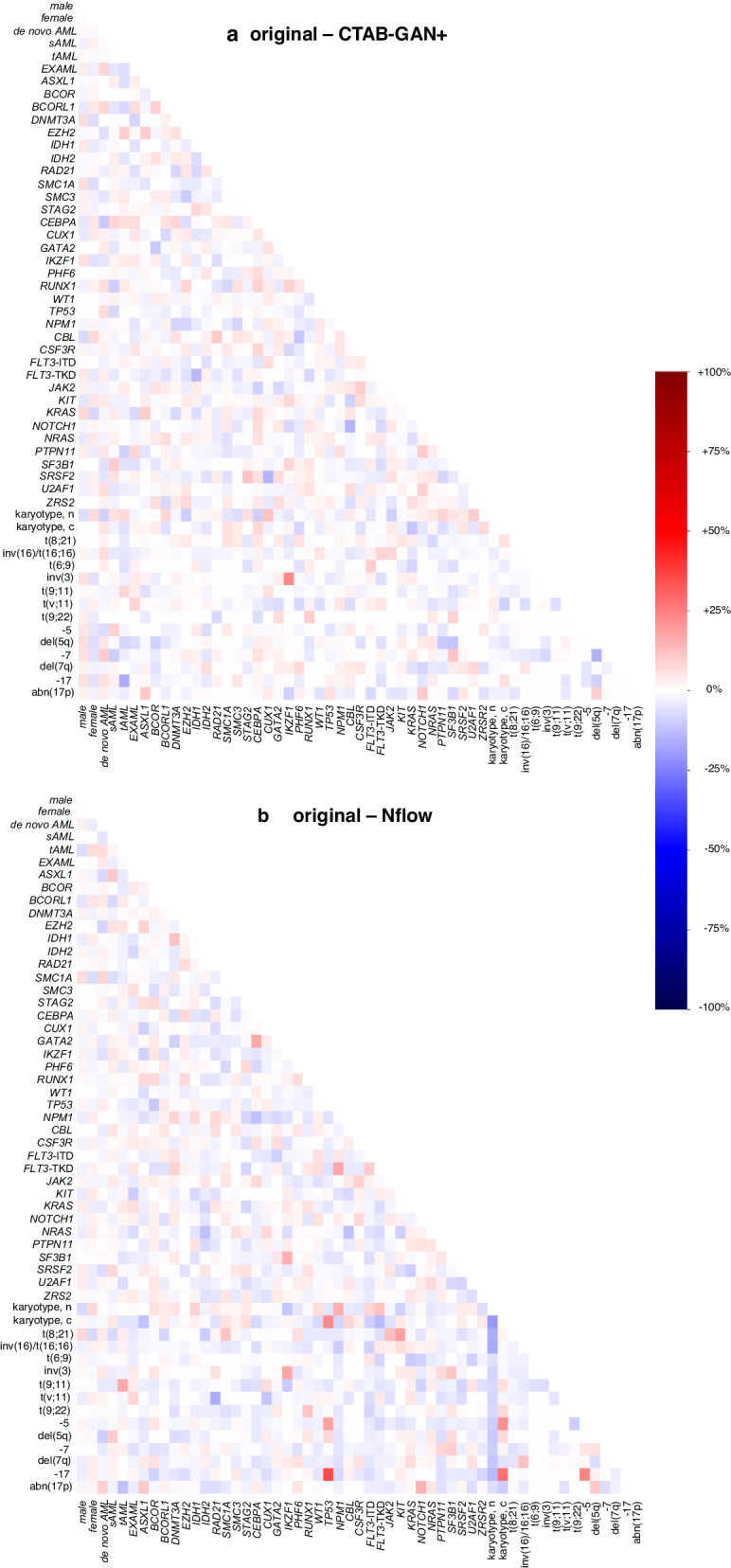
Heatmaps for relative differences of genetic and clinical associations. The differences in co-occurrences of genetic alterations as well as clinical variables are plotted. Relative increases (red) or decreases (blue) are displayed on a scale from −100% to +100%. The overlap between the original cohort and CTAB-GAN+ (**a**), as well as original and NFlow (**b**) showed high congruency. Increases or decreases in co-occurring genetic alterations were commonly found to affect alterations with low frequency in the original cohort.

Fifty molecular and cytogenetic alterations were included in generating synthetic patients. Figure 1 displays the distribution of these alterations across the original and synthetic cohorts (absolute numbers and *p*-values are provided in Supplementary Table 3). These alterations encompass genes that code for epigenetic regulators (Fig. 1a), the cohesin complex (Fig. 1b), transcription factors (Fig. 1c), *TP53* and *Nucleophosmin 1* (Fig. 1d), signaling factors (Fig. 1e), components of the spliceosome (Fig. 1f), and cytogenetic aberrations with established impact on patient outcome (Fig. 1g). Overall, the rates of alterations in both synthetic cohorts were in a plausible range with a few deviations from the original cohort of high statistical significance, such as NFlow-generated frequencies of *BCORL1, DNMT3A, PHF6*, and *ZRSR2*, as well as CTAB-GAN+-generated frequencies of *CUX1* and *GATA2* while the remainder of alterations showed only negligible differences. Aside from the frequency per individual alteration, the co-occurrences of alterations play an important role in disease biology, which should be also captured in high-quality synthetic data. Figure 2 shows the relative differences between the original cohort and CTAB-GAN+ (Fig. 2a) and NFlow (Fig. 2b) regarding co-occurring mutations. We found high congruencies for co-occurrences compared to the original cohort, while deviations were commonly found in alterations that had a low frequency in the original cohort. Reducing the degree of these discrepancies likely requires the generation of combinatorial features at the training stage which in turn vastly expands the feature space and destabilizes model training given the limited number of training samples^10^.

### Synthetic cohorts match real patients in outcome and survival analysis

**Fig. 3:**
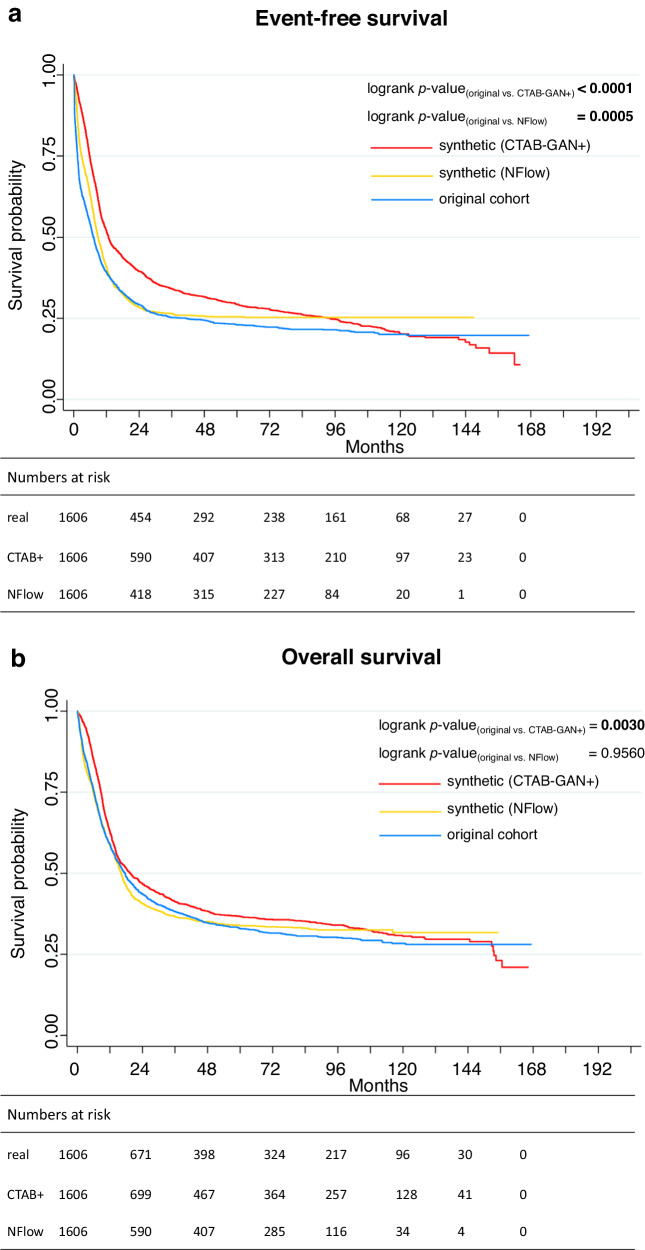
Comparison of survival curves between original and synthetic cohorts. Event-free survival (EFS) deviated significantly from the original cohort for both synthetic cohorts (**a**). For the NFlow-generated cohort, there was no significant deviation from the original distribution for overall survival (OS), while the CTAB-GAN + -generated cohort again differed significantly (**b**). Interestingly, while the survival curve for CTAB-GAN+ displays a plausible curve up until ten years of follow-up, the curve shows no stabilization of survival rates in the end as the original cohort does. Contrastingly, the survival curve for NFlow shows an overall plausible course, however, NFlow tends to overtly censor patients after two years of follow-up.

During model deployment, we compared whether optimizing for EFS or OS yielded better results. We found that using EFS directly during optimization led to inferior fidelity in the synthetic data. Hence, EFS had to be modeled indirectly to ensure a robust relation between both time-to-event variables. This effect can be seen as arising from the complex interplay between overall survival (OS) and event-free survival (EFS) within the generative networks, which appear to struggle in accurately representing two time-to-event variables at the same time. Median follow-up for the original cohort was 89.5 months (95%-CI: 85.5–95.4). The synthetic cohorts had a median follow-up of 91.3 months (CTAB-GAN+, 95%-CI: 84.8-98.0) and 74.3 months (NFlow, 95%-CI: 70.9–77.4). Tables 2, 3 show a detailed comparison of patient outcome between the original and both synthetic cohorts. For CR rates, we found no significant differences between the original (70.7%) and both synthetic cohorts (CTAB-GAN+: 73.7%; NFlow: 69.1%). Median EFS in the original cohort was 7.2 months while both CTAB-GAN+ with 12.8 months and NFlow with 9.0 months deviated with high significance. This effect can arguably be attributed to both CR rate and OS being included in hyperparameter tuning, while EFS was exempt from hyperparameter tuning. Kaplan-Meier analysis nevertheless showed a plausible representation of the survival curves for both synthetic cohorts regarding EFS (Fig. 3a). Median OS for the original cohort was 17.5 months while the CTAB-GAN+ cohort had a median OS of 19.5 months (*p* < 0.0001) and NFlow of 16.2 months (*p* = 0.055). Kaplan-Meier analysis (Fig. 3b) showed similar behavior of survival curves as for EFS. This was also evident with regard to usability metrics for synthetic survival data introduced by Norcliffe et al.^11^: We found both CTAB-GAN+ and NFlow to score high in our test set with normalized performance results (+1 is optimal representation, 0 is inadequate representation, Supplementary Table 1). First, we evaluated performance metrics for OS. Kaplan-Meier-Divergence, i.e. the degree to which survival curves of synthetic and real data differ, was low for both synthetic data sets (CTAB-GAN+: 0.97, NFlow: 0.98). Neither model showed overt optimism or overt pessimism in representing survival data (CTAB-GAN+: 0.98, NFlow: 0.99). Short-sightedness, i.e. failure to predict beyond a certain time point, was also low for both models, however slightly favoring CTAB-GAN+ over NFlow (CTAB-GAN+: 0.99, NFlow: 0.93) arguably corresponding to the censoring tendency of NFlow. For EFS, survival performance metrics were similar to OS (Supplementary Table 1) with a Kaplan-Meier-Divergence score of 0.94 and 0.96 for CTAB-GAN+ and NFlow, respectively. For EFS, both models showed low short sightedness (CTAB-GAN+: 0.98, NFlow: 0.88) and low optimism (CTAB-GAN+: 0.96, NFlow: 0.97). Still, visually the survival curve for EFS for both models was irregular as towards the end of the follow-up period there was still no stabilization of survival (Fig. 3). Notably, the number of patients with very long EFS or OS, i.e. over five years, was better matched by NFlow than CTAB-GAN+ compared to the original cohort (Supplementary Table 4).

**Table 2:** Comparison of patient outcomes between the original and synthetic cohort

	original cohort	CTAB-GAN +	NFlow
CR after induction therapy, *n* (%)	1135 (70.7)	1184 (73.7)	1110 (69.1)
OR	2.41	2.81	2.24
[95%-CI]	[2.16–2.68]	[2.51–3.14]	[2.01–2.49]
*p*-value		0.059	0.356
Median EFS, months (IQR)	7.2 (6.5–8.0)	12.8 (11.8–14.1)	9.0 (8.3–9.7)
HR	1.36	0.74	0.87
[95%-CI]	[1.25–1.47]	[0.68–0.80]	[0.80–0.94]
*p*-value		**<0.0001**	**<0.0001**
Median OS, months (IQR)	17.5 (15.7–19.2)	19.5 (15.7–19.2)	16.2 (15.7–19.2)
HR	1.14	0.88	1.00
[95%-CI]	[1.04–1.24]	[0.81–0.96]	[0.92–1.09]
*p*-value		**<0.0001**	0.055

Logistic regression and Cox proportional hazard models were used to obtain odds ratios (OR) for achievement of complete remission (CR) and hazard ratio (HR) with corresponding 95%-confidence intervals (95%-CI). Boldface indicates statistical significance (*p* < 0.05). *p*-values are calculated using two-sample comparisons between each of the synthetic cohorts and the original cohort for reference. *n* number.

### Synthetic data captures risk associations of individual variables for explorative analyses

In order to be useful for explorative analyses, synthetic data needs to recapitulate risk associations of individual variables. The ELN2022 recommendations represent one of the most widely used guidelines for risk stratification^12^. Hence, previously established markers of favorable (normal karyotype, t(8;21), inv(16) or t(16;16) mutations of *NPM1*, *CEBPA*-bZIP in frame mutations), intermediate risk (*FLT3*-ITD, t(9;11)), or adverse risk (complex karyotype, −5, del(5q), −7, −17, mutations of *TP53, RUNX1, ASXL1*), and age were evaluated using univariable analyses per cohort for their impact on achievement of CR, EFS, and OS. All effects for achievement of CR, EFS, and OS showed the same directionality – favorable affects in the original cohort were also favorable in synthetic cohorts and vice versa – and significance – effects that were significant in the original cohort were also significant in synthetic cohorts and vice versa (except for del(5q) being significantly associated with failure to achieve CR in the original cohort while this effect turned out to be non-significant in the NFlow-generated cohort). Importantly, no inverse effects – a variable that would be favorable in the original cohort would be adverse in a synthetic cohort or vice versa – were observed. Detailed outcomes per variable are reported for CR (Supplementary Table 5), EFS (Supplementary Table 6), and OS (Supplementary Table 7).

### Synthetically generated cohorts safeguard real patient data and prohibit re-identification

Privacy conservation was measured by: (i) number of exact matches between original and synthetic cohorts, (ii) a privacy leakage coefficient based on Hamming distance, and (iii) absolute Hamming distances showing the number of variables to be altered per synthetic patient to match a real patient. First, for both synthetic data sets the number of exact matches compared to the original cohort was zero. Second, the average minimum distances compared between datapoints in training and test sets were similar for the original cohort, as well as synthetic data from both CTAB-GAN+ and NFlow (Table 3). The privacy leakage coefficient – the quotient of Hamming distances between synthetic to test divided by synthetic to training data where small values (<0.05) indicate a small difference between the distances of synthetic data to training and test data, and therefore, indicate no privacy breach – was very low for both CTAB-GAN+ and NFlow (Table 3). This signals a low likelihood of re-identification for both synthetic datasets. Third, the median number of variables that would have to be altered to assign a synthetic patient to a training set patient was nine for both CTAB-GAN+ and NFlow.

**Table 3:** Hamming distances for privacy conservation

	CTAB-GAN+	NFlow	Original cohort
**Absolute Hamming distances**			
Average min. distance train	8.7034	9.3474	8.2524
Average min. distance test	8.8587	9.4117	8.2224
Median distance train	9	9	8
Median distance test	9	9	8
**Relative Hamming distances**			
privacy leakage coefficient	0.0178	0.0069	

Hamming distances were used to measure the distance between two points within and between equally sized subsets of training (four sets of 20%) and test data (20%). The median distance represents the number of variables that have to be altered (and matched exactly) to fit a real patient. A threshold for the privacy leakage coefficient of 0.05 for relative distances was set where values above 0.05 signal potential privacy breaches. Both synthetic data sets fell well below the 0.05 threshold signaling larger distances between synthetic and training data, which make a re-identification of training set patients unlikely.

## Discussion

Synthetic data provide an attractive solution to circumvent issues in current standards of data collection and sharing. These issues encompass first and foremost the time- and cost-intensive data collection process that usually involves enrollment of patients in prospective clinical trials presenting ever-increasing costs both regarding funding and time until completion, as well as ethical concerns inherent in clinical research with human subjects^3,4^. The prospect of using synthetic data as a kind of control group in prospective trials while effectively alleviating the need to enroll a larger number of patients and cutting costs bears the question of how closely such synthetic control arms match real-world cohorts. We used two generative AI technologies, a state-of-the-art GAN, CTAB-GAN + , and NFlow, to mimic the distribution of patient variables from four different previously conducted prospective multicenter trials including a total of 1606 patients with AML. Both models demonstrated high performance in previously established evaluation metrics that assess fidelity and usability of synthetic tabular data^9,11^. Generative models typically aim at reconstructing a given distribution. Nevertheless, changes in the distribution across the follow-up period in time-to-event data may, however, not be adequately captured using currently available models for tabular data generation. Hence, model architectures should be designed to also handle such distributional shifts over the follow-up period and improved metrics are needed for synthetic time-to-event analysis. This is especially pertinent as current synthetic survival metrics may not fully capture the nuances of long-term event prediction in survival analysis, particularly in the tail end of the survival curves. Such discrepancies underscore the need for additional or refined metrics that can better assess the accuracy and reliability of synthetic data in reflecting the prolonged survival trends. Further, multiple time-to-event endpoints may be of relevance in the context of clinical research. Model architectures need to be developed to simultaneously optimize for more than one time-to-event target variable. Currently, the design of synthetic cohorts may rely on selecting one target variable of interest to optimize for (in our case OS) and evaluate results for other outcomes. The comparison of distributions per variable between original and real data further showed close resemblances. Notably, even for statistically significant deviations from the original cohort, differences in effect sizes (e.g. age difference, difference in rates of occurrence for genetic alterations etc.) were often small. Inherent to hypothesis testing with such large sample sizes, even clinically irrelevant deviations can yield statistically significant differences. Importantly, inter-variable relationships were conserved in synthetic data: In univariable analyses both effect direction and statistical significance was well captured by both generative models effectively enabling explorative investigations in such data sets. Data sets of lower dimensionalities and comparable or even larger sample sizes, i.e. with a smaller feature space (fewer patient variables), may also allow for combinatorial variables to be generated and evaluated.

Once real data is obtained, privacy concerns often inhibit public access and thus impede data sharing and third-party hypothesis testing. Frequently used practices range from de-identifying or anonymizing data to more advanced computational approaches. De-identification or anonymization (e.g. removing names and birth dates), as well as adding artificial noise to the original data have recently been proven to be unsafe in terms of guarding privacy as reidentification attacks can successfully unveil patients’ identity^13–15^. Computational advances in both federated^16^ and swarm learning^17^ where machine learning models are trained across multiple locations and only either models or weights are shared rather than the data itself provide a viable alternative. Nevertheless, these technologies are vulnerable to data reconstructions, e.g. via data leakage from model gradients^18–20^. Inherent to synthetic data generation in terms of privacy safeguards is a trade-off between usability and privacy where an increase in each negatively affects the other^21^. Ideally, synthetic data should not be re-identifiable but at the same time closely match the original distributions. Zero exact matches were observed in our synthetic cohorts. Additionally, Hamming distances showed that reconstruction of original training samples is highly unlikely given the number of variables per synthetic patient that would have to be altered in order to match a training cohort patient.

The generation of synthetic data is, as all machine learning models are, fundamentally limited by the data that the model is trained on. This implies that external users should be aware of the properties of the training data that went into the generation of a synthetic data set in order to either select the right data set for their research question or vice versa, adapt the research question to the available data. It is therefore important to note, that patients in our trials have all been treated with intensive anthracycline-based therapy and largely stem from a Middle-European ethnic background. Hence, our generated synthetic AML data sets may not fully capture features of other populations let alone other treatment modalities, such as less intensive therapy or targeted agents. Treatment protocols in the trials used to generate synthetic patients in this study are all intensive anthracycline-based chemotherapy regimens. A further stratification of cohorts into individual treatment arms of the respective studies or according to transplantation status and a generation of synthetic patients based thereon was limited by the individual sample sizes of investigational and control arms as neural networks commonly require large data sets for robust training. One of the trials used for data generation, SORAML^22^, added sorafenib to the investigational cohort of which 110 patients have been included in model training. Notably, sorafenib did not affect CR rate or OS in the original study^22^. While sample subdivision likely represents a minor issue in the context of intensive chemotherapy-based regimens, it is acknowledged that for targeted agents, individual cohort generation is essential to adequately capture different mechanisms of action. To obtain large data sets for individual targeted agents, international multicenter collaboration will likely be necessary. The incorporation of these modalities will be addressed in future works. Since ML models thrive on large and diverse data sets, synthetic data generation from medical records is caught in a paradoxical loop: Available data is sparse, synthetic data can potentially accommodate for sparse available real data, synthetic data requires large and diverse sets of real data to meaningfully represent the population^23^. Therefore, the generation of synthetic data is likely more robust, if training data from large multicenter cohorts is used. An additional use case representing an essential data source can be devoted to real-world data. Differences in patient selection for clinical trials and patients in real-world settings may impede the generalizability of clinical trial findings to everyday practice^24^. Ideally, synthetic data generation would therefore also include patients from real-world settings. Still, the availability of patient features (such as comprehensive genetic information) may not be given in real-world data as it is in clinical trials. A homogenization of synthetic data from both sources at the same time may therefore be limited to patient features available from real-world settings. Hence, generation of distinct trial and real-world synthetic cohorts may be preferable for comparative analyses, i.e. comparing a real investigational arm to both a synthetic trial-based and synthetic real-world-based control.

Nonetheless, the availability of synthetic data promises a democratization of clinical research. In similar efforts regarding synthetic cancer patient data, Azizi et al.^25^ and D’Amico et al.^26^ explored synthetic data generation in cancer. Azizi et al.^25^ used data from a previously conducted clinical trial in colorectal cancer to generate synthetic data using conditional decision trees. Focusing on myelodysplastic neoplasms (MDS), D’Amico et al.^26^ used a conditional Wasserstein tabular GAN to generate synthetic MDS patients from the GenoMed4All database. Both groups conclude the feasibility of either method to generate synthetic data that closely resemble the original data distributions and provide access to their synthetic data. Publicly available synthetic data sets have increasingly demonstrated their value in advancing public health, for example, synthetic data from the UK primary care to evaluate machine learning models in healthcare^27^, model US-wide diseases of high morbidity^28^, construct databases for healthcare cost modeling in Medicare and Medicaid^29^, model mortality during the Covid19-pandemic^30^, or to assess community-specific exposure and risk^31^ for policy making. The advent of synthetic data in healthcare requires community-accepted guidelines^32^ to promote fairness^33^ and safety^34^. Different approaches to synthetic data generation and applications have been recently reviewed by Gonzales et al.^35^, Hernandez et al.^36^, Murtaza et al.^37^, or Jacobs et al^38^. These studies may alleviate a common gatekeeping mechanism of costly data collection efforts that are often restricted to large well-funded medical centers. Further, this also extends to cross-domain applications involving medical data, e.g. the training of a ML model by a third party that requires large sets of training data.

**Fig. 4:**
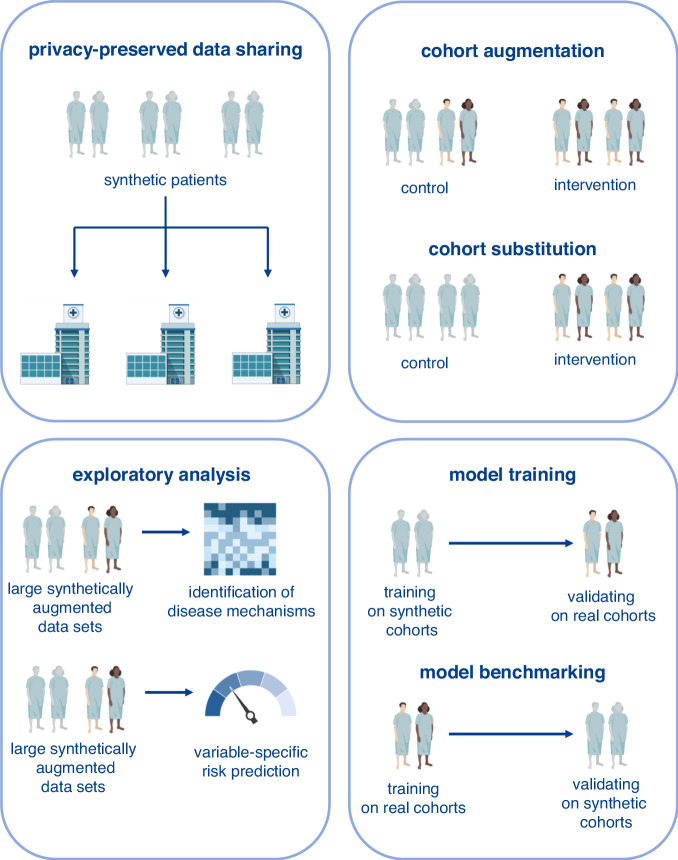
Use cases of synthetic data. Privacy conservation of synthetic data alleviates concerns of sharing identifying patient data. This enables simplified data sharing with the broader scientific community. Large publicly available data sets enable exploratory analysis of the synthetic data itself or by using synthetic data to augment proprietary data sets. This may encompass analyses of the genetic landscape of AML or the evaluation of the impact of specific alterations on patient risk. Further, training machine learning models requires large data sets. Training on publicly available synthetic data and validating on real data sets or vice versa, training on real data and using synthetic data as a benchmarking set for validation may foster the development of more robust machine learning models. Lastly, augmenting clinical trial cohorts with synthetic data or even substituting control cohorts with synthetic data entirely could re-shape prospective clinical trial designs. This, however, requires external validation and diligent regulatory oversight prior to implementation.

We here provide a large data set of a rare malignant entity with comprehensive patient-level information on clinical, laboratory, cytogenetic, molecular, and outcome variables with a variety of potential use cases (Fig. 4). First, by sharing this dataset with the scientific community, we aim to foster research efforts that require large such large datasets, for example, exploratory analysis to identify variable-specific behavior (e.g. association of a certain molecular variable with patient outcomes), to train, test, and validate external machine learning models (e.g. classifiers to predict patient outcomes), or to augment existing cohorts. The latter may at some point include the augmentation of clinical trial control cohorts, however, the limitations noted above have to be considered and communicated with the responsible ethics committees. Future work in evaluating the augmentation or substitution of control cohorts of large prospective clinical trials with synthetic data is needed to establish synthetic benchmark sets for widespread usage. The advent of synthetic data in clinical trial settings requires additional stringent regulatory oversight^39^. The current lack of regulatory guidance not only disables potential uses of synthetic data in clinical trials, but also enables regulatory blind spots for predatory actors to misuse synthetic healthcare data since current legal frameworks of data protection such as the General Data Protection Regulation (GDPR) and the Health Insurance Portability and Accountability Act (HIPAA) fall short in addressing potential issues regarding synthetic data^39,40^. Given the pace with which AI currently develops, there is a widening gap between how fast technologies are devised and how much later regulatory agencies set legal boundaries for the technologies’ safe implementation^41^. This likely requires a multifaceted regulatory approach involving anticipation of technological developments and proactive regulation of potential use cases^41^. Moreover, regulatory agencies will need to foster active dialogue between all involved stakeholders in synthetic healthcare data generation including first and foremost patients and patient advocacy groups, but also healthcare providers, legislators, legal experts, researchers in academia and industry, as well as software developers and engineers building generative models.

In summary, we demonstrate the feasibility of two different technologies of generative AI to create synthetic clinical trial data that both closely mimic disease biology and clinical behavior, as well as conserve the privacy of patients in the training cohort. Generating such large synthetic data sets based on multicenter clinical trial training data holds the promise of enabling a new kind of clinical research improving upon data accessibility, while ameliorating current hindrances in data sharing.

## Methods

### Patient data

Multimodal clinical, laboratory, and genetic data (Supplementary Table 8) were obtained from 1606 patients with non-M3 AML that were treated within previously conducted multicentric prospective clinical trials of the German Study Alliance Leukemia (SAL; AML96 [NCT00180115]^42^, AML2003 [NCT00180102]^43^, AML60+ [NCT00180167]^44^, and SORAML [NCT00893373]^22^). Supplementary Table 9 shows an overview of trial protocols. Eligibility was determined upon diagnosis of AML, age ≥18 years, and curative treatment intent. All patients gave their written informed consent according to the revised Declaration of Helsinki^45^. All studies were previously approved by the Institutional Review Board of the Technical University Dresden. Complete remission (CR), event-free survival (EFS), and overall survival (OS) were defined according to the revised ELN criteria^12^. Biomaterial was obtained from bone marrow aspirates or peripheral blood prior to treatment initiation. Sample collection, biobanking, use of samples and clinical information as well as analysis of individual patient data was carried out under the auspices of the SAL bioregistry. All these activities carried out for the purpose of retrospective research such as this study on previously acquired data were approved by the Institutional Review Board of the Technical University Dresden (EK 98032010). Next-Generation Sequencing (NGS) was performed retrospectively using the TruSight Myeloid Sequencing Panel (Illumina, San Diego, CA, USA). Pooled samples were sequenced paired-end and a 5% variant allele frequency (VAF) mutation calling cut-off was used with human genome build HG19 as a reference as previously described in detail^46^. Additionally, high resolution fragment analysis for *FLT3*-ITD^47^, *NPM1*^48^, and *CEBPA*^49^ was performed as described previously. For cytogenetics, standard techniques for chromosome banding and fluorescence-in-situ-hybridization (FISH) were used.

### Generative models

In our study, we used two state-of-the-art generative models exhibiting two fundamentally different concepts of data generation:

i) CTAB-GAN + ^50^ builds upon the Generative Adversarial Network (GAN)^51^ architecture, consisting of two interlinked neural networks - the generator and the discriminator. These are jointly trained in an adversarial manner. The generator’s goal is to produce synthetic data that appears realistic, starting from random noise. In parallel, the discriminator seeks to differentiate between real and synthetic samples created by the generator. The training continues until the discriminator is no longer able to reliably distinguish real data from synthetic, indicating that the generator has successfully approximated the distribution of the real data.

ii) Normalizing Flows (NFlow)^52^ presents an alternative approach for synthesizing data from complex distributions. This comprises a sequence of invertible transformations, starting from a simple base distribution. Each transformation, or ‘flow’, gradually modifies this base distribution into a more complex one that better mirrors the actual data. Importantly, these transformations are stackable, meaning they can be applied successively to incrementally increase the complexity of the modeled distribution. All parameters defining these flows are learned directly from the data, allowing the model to accurately capture the underlying data distribution. Note, that we used a modification of NFlow for survival data provided by the Synthcity^53^ software framework.

No imputation of missing data was performed in the original data set, thus both final synthetic data sets also contain missing data to adequately represent real-world conditions. For model training, missingness was denoted as an additional state per variable (for example: 1 = present, 0 = absent, na = missing). Hence, for binary features a trinary model output was possible (0, 1, na). Supplementary Table 10 denotes the number of missing values. Hyperparameter tuning was performed using the Optuna framework allowing both generative models to capture the best possible representation of the original data. During the development process, we initially modeled EFS directly but observed unrealistic time-to-event data where EFS sometimes surpassed OS. To address this, we shifted to an indirect approach, modeling the difference between OS and EFS instead of EFS directly. Subsequently, hyperparameters were tuned for CR, OS, and the difference between EFS and OS. This led to a more robust and consistent representation of both OS and EFS simultaneously without the logical flaws (EFS > OS) that we saw before.

### Evaluation of synthetic data performance

To assess the fidelity und usability of synthetic data, previously proposed evaluation metrics were used to provide a comprehensive overview of model performance. In particular, Basic Statistical Measure, Regularized Support Coverage, and Log-transformed Correlation Score were used to evaluate the fidelity of the data in general via our implementation based on the descriptions by Chundawat et al.^9^. The second set of metrics – Kaplan-Meier-Divergence, Optimism and Short-Sightedness - was previously introduced by Norcliffe et al.^11^ for synthetic survival data. NFlow was implemented in Synthcity^53^ where the time-to-event variable of interest was set to OS. For improved comparability, performance metrics were normalized on a scale from 0 (inadequate representation of original data) to 1 (optimal representation). An overview of the underlying methodologies of these metrics is provided in Supplementary Table 11. For detailed information, we refer the interested reader to the original publications^9,11^.

### Assessment of privacy conservation

To assess potential privacy implications of synthetic data, we customized the method proposed by Platzer and Reutterer^54^ to accommodate for smaller sample sizes. We partitioned the original training data (80% of total) into four subsets, matching the size of the test dataset (20%) for balanced comparisons (Supplementary Fig. 2). Calculations were performed using Hamming distance^55^ for categorical features. Numerical variables were binned (*n* = 10 bins each) and thereby categorized to enable Hamming distance calculations. Given the nature of the Hamming distance metric, the average minimum distance effectively denotes the number of variables that would need to be altered for a synthetic patient to match a real patient. We compared the average distances of the synthetic data to the training (syn → train) and test sets (syn → test). The relationship between both can be expressed as a coefficient for each synthetic data set compared to training and test set:

$${privacy}\,{leakage}\,{coefficient}=\frac{{syn}\to {test}}{{syn}\to {train}}-1$$

By analyzing whether the synthetic data is closer to the training set compared to the test set, we can assess whether the synthetic data is overly representative of the training data, thereby posing potential privacy concerns. If the average distances from the synthetic data to the training and test data are equally small, the privacy leakage coefficient will also be small. The lower the privacy leakage coefficient, the lower the likelihood of re-identification for patients in the training set. We assumed that values above 0.05 signal potential privacy breaches, as they suggest the synthetic data is substantially closer to the training set than to the test set. Conversely, values below 0.05 denote a favorable privacy safeguard, signaling similar distances between the training and test sets. Additionally, the number of exact subject matches between the synthetic and original cohorts was determined.

### Statistical analysis

Pairwise analyses were conducted between the original and both synthetic data sets. Normality was assessed using the Shapiro-Wilk test. If the assumption of normality was met, continuous variables between two samples were analyzed using the two-sided unpaired t-test. If the assumption of normality was violated, continuous variables between two samples were analyzed using the Wilcoxon rank sum (syn. Mann-Whitney) test. Fisher’s exact test was used to compare categorical variables. Univariate analyses for binary outcomes (CR rate) were carried out via logistic regression to obtain odds ratios (OR) and 95% confidence intervals (95%-CI). Time-to-event analyses (EFS, OS) were carried out using Cox proportional hazard models to obtain hazard ratios (HR) and 95%-CI. Kaplan-Meier analyses were performed for time-to-event data (EFS, OS) and corresponding log-rank tests are reported. Median follow-up time was calculated using the reverse Kaplan-Meier method^56^. All tests were carried out as two-sided tests. Statistical significance was determined using a significance level α of 0.05. Statistical analysis was performed using STATA BE 18.0 (Stata Corp, College Station, TX, USA).

### Reporting summary

Further information on research design is available in the Nature Research Reporting Summary linked to this article.

### Supplementary information

Supplements

Reporting Summary

## Data Availability

The synthetic data^57^ sets generated and analyzed for the purpose of this study are publicly available at https://zenodo.org/record/8334265 or via 10.5281/zenodo.8334265.

## Code availability

The underlying code generated for the purpose of this study is publicly available at https://github.com/waldemar93/synthetic_data_pipeline.
