# Supplementary material for: Mimicking clinical trials with synthetic acute myeloid leukemia patients using generative artificial intelligence
Source: NPJ Digit Med. 2024 Mar 20;7:76. doi: 10.1038/s41746-024-01076-x (PMC10954666; doi:10.1038/s41746-024-01076-x)
Supplement: Supplementary file 1 — Supplements [file 41746_2024_1076_MOESM1_ESM.pdf]

Supplements

performance metric	original cohort	CTAB-GAN+	NFLOW
Log-transformed Correlation Score	0.58	0.75	0.74
Regularized Support Coverage	0.93	0.95	0.97
Basic Statistical Measure	0.95	0.91	0.92
Optimism (OS)	1.00	0.97	0.98
Optimism (EFS)	0.99	0.96	0.97
Kaplan-Meier Divergence (OS)	1.00	0.98	0.99
Kaplan-Meier Divergence (EFS)	0.99	0.94	0.96
Short-Sightedness (OS)	0.94	0.99	0.93
Short-Sightedness (EFS)	0.94	0.98	0.88

Supplementary Table 1 Performance evaluation of both generative models. Previously proposed performance metrics for tabular synthetic data (introduced by Chundawat et al.[9] and Norcliffe et al.[11]) were used to evaluate model performance. All metrics are scaled from 0 (inadequate representation of original data) to 1 (optimal representation).

Trial	train (n)	test (n)	*p*
AML96	766	183	0.567
AML60+	39	6	1.000
AML2003	146	42	0.380
SORAML	182	47	0.788
AML registry	158	37	0.923

Supplementary Table 2 Patient distribution according to source trial between training and test set.

		original cohort	CTAB-GAN+	*p*	NFlow	*p*
number of patients		1606	1606		1606	
**molecular genetics, n(%)**						
epigenetic	*ASXL1*	126 (7.9)	161 (10.0)	0.035	113 (7.0)	0.420
	BCOR	76 (4.7)	78 (4.9)	0.934	72 (4.5)	0.801
	BCORL1	60 (3.7)	59 (3.7)	1.000	182 (11.3)	0.000
	DNTM3A	458 (28.5)	413 (25.7)	0.081	547 (34.0)	0.001
	EZH2	63 (3.9)	75 (4.7)	0.339	63 (3.9)	1.000
	IDH1	149 (9.3)	156 (9.7)	0.718	149 (9.3)	1.000
	IDH2	227 (14.1)	214 (13.3)	0.538	278 (17.3)	0.015
	TET2	311 (19.4)	313 (19.5)	0.964	308 (19.2)	0.929
transcription	*CEBPA*	257 (16.0)	323 (20.1)	0.002	268 (16.7)	0.503
	CEBPA. biallelic	92 (5.7)	132 (8.2)	0.001	89 (5.5)	0.878
	CEBPA-TAD	37 (2.3)	50 (3.1)	0.102	24 (1.5)	0.194
	CEBPA-bZIP	144 (9.0)	206 (12.8)	0.851	127 (7.9)	0.001
	CUX1	44 (2.7)	86 (5.4)	<0.001	48 (3.0)	0.751
	GATA2	97 (6.0)	159 (9.9)	<0.001	109 (6.8)	0.428
	IKZF1	45 (2.8)	39 (2.4)	0.581	37 (2.3)	0.434
	PHF6	52 (3.2)	65 (4.1)	0.258	109 (6.8)	0.000
	RUNX1	147 (9.2)	159 (9.9)	0.509	156 (9.7)	0.629

	WT1	118 (7.4)	80 (5.0)	0.007	117 (7.3)	1.000
signaling	*CBL*	32 (2.0)	44 (2.7)	0.201	28 (1.7)	0.696
	CSF3R	29 (1.8)	44 (2.7)	0.097	33 (2.1)	0.701
	FLT3-ITD	349 (21.7)	347 (21.6)	1.000	363 (22.6)	0.496
	FLT3-TKD	62 (3.9)	53 (3.3)	0.633	94 (5.9)	0.004
	JAK2	18 (1.1)	23 (1.4)	0.530	22 (1.4)	0.634
	KIT	79 (4.9)	97 (6.0)	0.187	75 (4.7)	0.804
	KRAS	85 (5.3)	115 (7.2)	0.034	77 (4.8)	0.573
	NOTCH1	32 (2.0)	39 (2.4)	0.472	43 (2.7)	0.242
	NRAS	249 (15.5)	305 (19.0)	0.010	198 (12.3)	0.011
	PTPN11	113 (7.0)	119 (7.4)	0.733	102 (6.4)	0.480
splicing	*SF3B1*	46 (2.9)	48 (3.0)	0.917	41 (2.7)	0.664
	SRSF2	102 (6.4)	101 (6.3)	1.000	138 (8.6)	0.019
	U2AF1	45 (2.8)	51 (3.2)	0.605	45 (2.8)	1.000
	ZRSR2	26 (1.6)	20 (1.3)	0.458	64 (4.0)	0.000
cohesin	*RAD21*	51 (3.2)	58 (3.6)	0.559	44 (2.7)	0.532
	SMC1A	23 (1.4)	26 (1.6)	0.774	22 (1.4)	1.000
	SMC3	18 (1.1)	31 (1.9)	0.083	37 (2.3)	0.014

	STAG2	88 (5.5)	69 (4.3)	0.141	132 (8.2)	0.003
other	*TP53*	114 (7.1)	100 (6.2)	0.358	115 (7.2)	1.000
	NPM1	501 (31.2)	507 (31.6)	0.819	508 (31.6)	0.674
**cytogenetics. n (%)**						
	normal karyotype	830 (51.7)	780 (48.6)	0.085	788 (49.1)	0.143
	complex karyotype	188 (11.7)	209 (13.0)	0.280	229 (14.3)	0.072
	t(8;21)	61 (3.8)	90 (5.6)	0.019	71 (4.4)	0.424
	inv(16) or t(16;16)	101 (6.3)	101 (6.3)	1.000	89 (5.5)	0.371
	t(6;9)	6 (0.4)	5 (0.3)	0.774	11 (0.7)	0.331
	inv(3) or t(3;3)	7 (0.4)	17 (1.1)	0.063	10 (0.6)	0.628
	t(9;11)	11 (0.7)	22 (1.4)	0.079	22 (1.4)	0.079
	t(v;11)	16 (1.0)	34 (2.1)	0.015	15 (0.9)	0.859
	t(9;22)	3 (0.2)	3 (0.2)	1.000	12 (0.8)	0.035
	-5	24 (1.5)	28 (1.7)	0.675	31 (1.9)	0.415
	del(5q)	18 (1.1)	23 (1.4)	0.530	20 (1.3)	0.871
	-7	71 (4.4)	83 (5.2)	0.364	92 (5.7)	0.108

del(7q)	16 (1.0)	15 (0.9)	0.859	36 (2.2)	0.007
-17	34 (2.1)	38 (2.4)	0.721	21 (1.3)	0.079
abn(17p)	6 (0.4)	13 (0.8)	0.166	18 (1.1)	0.022

Supplementary Table 3 Distribution of molecular and cytogenetic alterations between the original and the synthetic cohorts. *p*-values are calculated using two-sample comparisons between each of the synthetic cohorts and the baseline cohort for reference. Abbreviations: number (n).

	**original cohort**	**CTAB-GAN+**	**NFLOW**
patients with OS > 5 years	362 (22.8%)	409 (25.5%)	343 (21.4%)
patients with EFS > 5 years	265 (16.7%)	356 (22.2%)	275 (17.1%)
patients with OS > 5 years AND EFS < 5 years	97 (6.1%)	53 (3.3%)	68 (4.2%)

Supplementary Table 4 Number of patients with very long event-free and overall survival.

	original	*p*	CTAB-GAN+	*p*	NFlow	*p*
age	0.94 [0.93-0.95]	<0.001	0.94 [0.93-0.95]	<0.001	0.95 [0.94-0.95]	<0.001
normal karyotype	1.98 [1.58-2.49]	<0.001	2.22 [1.75-2.81]	<0.001	1.50 [1.20-1.88]	<0.001
complex karyotype	0.40 [0.29-0.54]	<0.001	0.39 [0.29-0.53]	<0.001	0.58 [0.43-0.77]	<0.001
inv(16) or t(16;16)	3.25 [1.76-5.99]	<0.001	1.82 [1.07-3.10]	0.028	2.73 [1.50-4.97]	0.001
t(8;21)	8.38 [2.61-26.89]	<0.001	3.37 [1.68-6.77]	0.001	3.20 [1.58-6.49]	0.001
t(9;11)	1.87 [0.40-8.69]	0.424	1.21 [0.45-3.31]	0.704	0.78 [0.32-1.87]	0.576
-5	0.13 [0.05-0.34]	<0.001	0.16 [0.07-0.36]	<0.001	0.24 [0.11-0.50]	<0.001
del(5q)	0.33 [0.13-0.83]	0.019	0.07 [0.02-0.21]	<0.001	0.67 [0.27-1.64]	0.378
-7	0.25 [0.15-0.41]	<0.001	0.24 [0.15-0.37]	<0.001	0.20 [0.13-0.32]	<0.001
-17	0.12 [0.05-0.27]	<0.001	0.10 [0.05-0.22]	<0.001	0.33 [0.14-0.79]	0.013
NPM1	2.49 [1.91-3.24]	<0.001	2.80 [2.11-3.70]	<0.001	1.69 [1.33-2.15]	<0.001
FLT3-ITD	1.79 [1.35-2.39]	<0.001	2.12 [1.55-2.91]	<0.001	1.41 [1.08-1.84]	0.011

CEBPA-bZIP (inframe)	8.12 [3.56-18.57]	<0.001	4.88 [2.47-9.67]	<0.001	3.56 [1.78-7.15]	<0.001
TP53	0.14 [0.09-0.22]	<0.001	0.17 [0.11-0.26]	<0.001	0.17 [0.11-0.26]	<0.001
RUNX1	0.30 [0.21-0.42]	<0.001	0.20 [0.14-0.28]	<0.001	0.54 [0.39-0.76]	<0.001
ASXL1	0.35 [0.25-0.51]	<0.001	0.42 [0.30-0.58]	<0.001	0.46 [0.31-0.68]	<0.001

Supplementary Table 5 Comparative univariable analyses for individual patient variables with respect to achievement of complete remission. Variables with previously demonstrated impact on patient outcome were analyzed using univariable logistic regression. Their odds ratio (OR) and 95%-confidence interval (square brackets) as well as corresponding *p*-values are reported per cohort. Except for del(5q) being significantly associated with failure to achieve CR in the original cohort while this effect turned out to be non-significant in the NFlow-generated cohort, all other effects were of the same directionality and statistical significance. Importantly, no variable showed an inverted effect (for example, a favorable marker turning unfavorable in a synthetic cohort).

	original	*p*	CTAB-GAN+	*p*	NFlow	*p*
age	1.03 [1.03-1.03]	<0.001	1.03 [1.03-1.03]	<0.001	1.03 [1.03-1.04]	<0.001
normal karyotype	0.82 [0.73-0.93]	0.001	0.85 [0.76-0.96]	0.008	0.82 [0.73-0.92]	0.001
complex karyotype	1.64 [1.39-1.93]	<0.001	1.68 [1.43-1.98]	<0.001	1.44 [1.23-1.69]	<0.001
inv(16) or t(16;16)	0.58 [0.44-0.74]	<0.001	0.43 [0.32-0.58]	<0.001	0.58 [0.44-0.77]	<0.001
t(8;21)	0.35 [0.21-0.52]	<0.001	0.38 [0.28-0.53]	<0.001	0.41 [0.29-0.58]	<0.001
t(9;11)	0.64 [0.30-1.34]	0.237	0.84 [0.50-1.42]	0.525	1.19 [0.74-1.92]	0.481
-5	3.53 [2.35-5.30]	<0.001	3.92 [2.68-5.72]	<0.001	2.51 [1.72-3.69]	<0.001
del(5q)	2.76 [1.73-4.41]	<0.001	3.40 [2.24-5.15]	<0.001	1.76 [1.10-2.80]	0.018
-7	2.82 [2.20-3.61]	<0.001	3.10 [2.47-3.90]	<0.001	2.97 [2.37-3.71]	<0.001
-17	3.32 [2.34-4.70]	<0.001	3.50 [2.42-4.84]	<0.001	1.97 [1.23-3.13]	0.004
NPM1	0.68 [0.60-0.77]	<0.001	0.77 [0.68-0.87]	<0.001	0.73 [0.64-0.82]	<0.001
FLT3-ITD	1.00 [0.88-1.15]	0.959	1.04 [0.91-1.20]	0.564	0.95 [0.83-1.09]	0.461

CEBPA-bZIP (inframe)	0.39 [0.28-0.54]	<0.001	0.44 [0.34-0.60]	<0.001	0.64 [0.47-0.88]	0.006
TP53	2.82 [2.31-3.44]	<0.001	3.34 [2.71-4.13]	<0.001	3.05 [2.50-3.73]	<0.001
RUNX1	1.88 [1.57-2.24]	<0.001	1.95 [1.63-2.31]	<0.001	1.76 [1.48-2.10]	<0.001
ASXL1	1.86 [1.54-2.25]	<0.001	1.62 [1.31-2.01]	<0.001	1.52 [1.28-1.81]	<0.001

Supplementary Table 6 Comparative univariable analyses for individual patient variables with respect to event-free survival. Variables with previously demonstrated impact on patient outcome were analyzed using univariable logistic regression. Their Hazard Ratio (HR) and 95%-confidence interval (square brackets) as well as corresponding *p*-values are reported per cohort. No discrepancies between effect direction and statistical significances of effects were found between the original and both synthetic cohorts were found.

	**original**	***p***	**CTAB-GAN+**	***p***	**NFlow**	***p***
age	1.04 [1.03-1.04]	<0.001	1.03 [1.03-1.04]	<0.001	1.04 [1.03-1.05]	<0.001
normal karyotype	0.80 [0.71-0.91]	0.001	0.78 [0.69-0.88]	<0.001	0.81 [0.71-0.92]	0.001
complex karyotype	1.72 [1.44-2.04]	<0.001	1.86 [1.58-2.20]	<0.001	1.42 [1.20-1.67]	<0.001
inv(16) or t(16;16)	0.52 [0.39-0.70]	<0.001	0.41 [0.29-0.57]	<0.001	0.56 [0.41-0.78]	<0.001
t(8;21)	0.33 [0.21-0.51]	<0.001	0.37 [0.26-0.53]	<0.001	0.41 [0.28-0.60]	<0.001
t(9;11)	0.60 [0.25-1.43]	0.247	0.88 [0.50-1.55]	0.655	1.22 [0.74-2.04]	0.436
-5	4.37 [2.90-6.57]	<0.001	4.30 [2.95-6.28]	<0.001	2.44 [1.66-3.57]	<0.001
del(5q)	2.32 [1.41-3.80]	0.001	3.22 [2.13-4.88]	<0.001	1.83 [1.14-2.96]	0.013
-7	2.79 [2.17-3.58]	<0.001	3.16 [2.41-3.97]	<0.001	2.77 [2.21-3.46]	<0.001

-17	3.68 [2.59-5.21]	<0.001	3.46 [2.49-4.79]	<0.001	1.91 [1.19-3.09]	0.008
NPM1	0.74 [0.65-0.85]	<0.001	0.72 [0.65-0.82]	<0.001	0.75 [0.65-0.86]	<0.001
FLT3-ITD	1.06 [0.92-1.22]	0.440	1.01 [0.87-1.18]	0.853	0.95 [0.82-1.10]	0.468
CEBPA-bZIP (inframe)	0.41 [0.28-0.59]	<0.001	0.42 [0.31-0.57]	<0.001	0.68 [0.48-0.96]	0.027
TP53	3.44 [2.81-4.21]	<0.001	3.65 [2.85-4.52]	<0.001	2.75 [2.24-3.37]	<0.001
RUNX1	1.82 [1.51-2.19]	<0.001	1.92 [1.61-2.30]	<0.001	1.69 [1.41-2.03]	<0.001
ASXL1	1.64 [1.35-2.01]	<0.001	1.55 [1.29-1.87]	<0.001	1.68 [1.36-2.08]	<0.001

Supplementary Table 7 Comparative univariable analyses for individual patient variables with respect to overall survival. Variables with previously demonstrated impact on patient outcome were analyzed using univariable logistic regression. Their Hazard Ratio (HR) and 95%-confidence interval (square brackets) as well as corresponding *p*-values are reported per cohort. No discrepancies between effect direction and statistical significances of effects were found between the original and both synthetic cohorts were found.

patient variable	data type
**demographic/clinical**	
age	continuous
sex	binary
AML status (de novo, sAML, tAML)	categorical
extramedullary manifestations	binary
**laboratory values**	
white blood cell count	continuous
hemoglobin level	continuous
platelet count	continuous
**outcome**	
achievement of CR	binary
EFS duration	continuous
EFS status	binary
OS duration	continuous
OS status	binary
**molecular genetics**	
ASXL1	binary
BCOR	binary
BCORL1	binary
DNTM3A	binary
EZH2	binary
IDH1	binary
IDH2	binary
TET2	binary
RAD21	binary
SMC1A	binary
SMC3	binary
STAG2	binary
CEBPA	binary
CEBPA-bZIP in frame	binary
CUX1	binary
GATA2	binary
IKZF1	binary
PHF6	binary
RUNX1	binary
WT1	binary
TP53	binary
NPM1	binary
CBL	binary
CSF3R	binary
FLT3-ITD	binary
FLT3-TKD	binary
JAK2	binary
KIT	binary
KRAS	binary
NOTCH1	binary
NRAS	binary
PTPN11	binary
SF3B1	binary
SRSF2	binary

U2AF1	binary
ZRSR2	binary
**cytogenetics**	
normal karyotype	binary
complex karyotype	binary
t(8;21)	binary
inv(16) or t(16;16)	binary
t(6;9)	binary
inv(3) or t(3;3)	binary
t(9;11)	binary
t(v;11)	binary
t(9;22)	binary
-5	binary
del(5q)	binary
-7	binary
del(7q)	binary
-17 or del(17p)	binary
abn(17p)	binary

Supplementary Table 8 Available patient variables included in synthetic data generation. Abbreviations: complete remission (CR), event-free survival (EFS), overall survival (OS), secondary acute myeloid leukemia (sAML), therapy-associated acute myeloid leukemia (tAML).

trial name	clinicaltrials.gov identifier	trial duration	protocol summary
AML96	NCT00180115	1996-2008	risk-adapted postremission treatment regarding allogeneic stem cell transplantation for high-risk AML and related allogeneic and autologous stem cell transplantation for standard-risk AML, and randomization between intermediate-dose and high-dose cytarabine within the first post-remission course
AML2003	NCT00180102	2003-2009	early allogeneic stem cell transplantation in post-induction aplasia for high-risk AML, factorial design with four therapy arms with two factors of two stages (intensified vs. standard therapy and cytarabine vs. cytarabine + mitoxantrone + amsacrin)
AML60+	NCT00180167	2005-2010	Patients ≥ 60 years, mitoxantron on day 1,2,3 + cytarabine on days 1,3,5,7 vs. DA 7+3
SORAML	NCT00893373	2011-2014	Standard therapy + sorafenib vs. standard therapy + placebo

Supplementary Table 9. Summary of trial regimens

**Variable**	**original (n)**	**original (%)**	**CTAB-GAN+ (n)**	**CTAB-GAN+ (%)**	**NFlow (n)**	**NFlow (%)**
EXAML	138	8,68%	157	9,78%	110	6,85%
AMLSTAT	18	1,13%	17	1,06%	73	4,55%
CEBPA	64	4,03%	69	4,30%	83	5,17%
FLT3-ITD	25	1,57%	34	2,12%	37	2,30%
FLT3-TKD	519	32,64%	592	36,86%	570	35,49%
NPM1	33	2,08%	37	2,30%	47	2,93%

Supplementary Table 10 Missing values. For two clinically assessed patient variables, extramedullary AML (EXAML) and AML status (AMLSTAT; de novo AML/sAML/tAML) values were missing from the original cohort. Additionally, for molecular alterations assessed via targeted sequencing, there were missing values. For all other variables, no missing values were present in the original data and therefore no missing values were generated by both models for these variables.

Performance metric	Explanation	Reference
**Basic Statistical Measure**	compares the mean, median, and standard deviation between numerical columns of both real and synthetic datasets to assess their similarity	Chundawat et al.
**Regularized Support Coverage**	quantifies the overlap in individual feature distributions between the original and synthetic datasets, ensuring both share similar support for each feature	Chundawat et al.
**Log-transformed Correlation Score**	evaluates the difference in correlation matrices between the original and synthetic datasets, which helps assess how well the synthetic data captures inter-feature relationships	Chundawat et al.
**Kaplan-Meier Divergence**	This metric calculates the mean absolute difference between the Kaplan-Meier survival curves of the synthetic and real data, measuring the overall match between the survival probabilities.	Norcliffe et al.
**Optimism**	This survival analysis metric measures the discrepancy in	Norcliffe et al.

	expected lifetimes between the synthetic and real data, as illustrated by their respective Kaplan-Meier survival curves. It quantifies the degree of over-optimism or over-pessimism in the synthetic data	
**Short-Sightedness**	This metric quantifies the extent to which models, as evaluated by Kaplan-Meier survival curves of synthetic data, fail to predict beyond a certain time horizon, capturing temporal limitations in the synthetic data	Norcliffe et al.

Supplementary Table 11 Performance metrics for fidelity and usability of synthetic data.

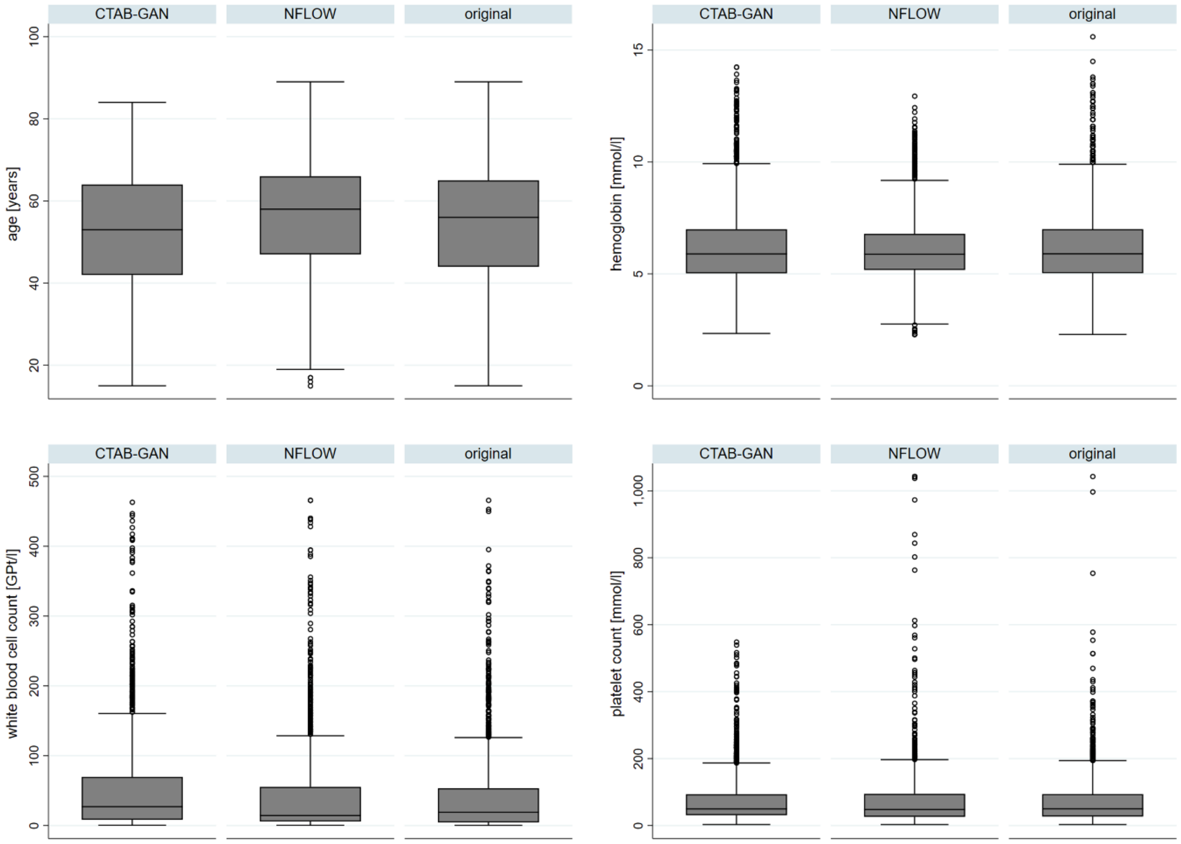

Supplementary Figure 1 Representation of continuous variables by CTAB-GAN+ and NFlow. Boxplot: bold horizontal line = median; box = interquartile range (IQR, i.e. 25th to 75th percentile); lower whisker = Q1 – 1.5 * IQR; upper whisker = Q3 + 1.5 * IQR; dots = outliers. In the original data set, the number of patients with outlier values for continuous variables was gradually decreased towards the upper end of the spectrum as more extreme outliers are less likely. This behavior was better represented by NFlow than by CTAB-GAN+. For white blood cell count, CTAB-GAN+ seemed to even out the outliers across the upper distribution range resulting in a statistically significant difference compared to the original cohort (Tab. 1) whereas outliers for Nflow were more in line with the original cohort. For platelet count, CTAB-GAN+ cut off outliers at the 600 GPt/l mark whereas NFlow came closer to matching the original distribution. For Hb, CTAB-GAN+, however, mimicked the original distribution better than NFlow which generated fewer outliers to the top of the distribution range. Notably, for age this behavior was not observed, arguably as the original data did not include extreme outliers.

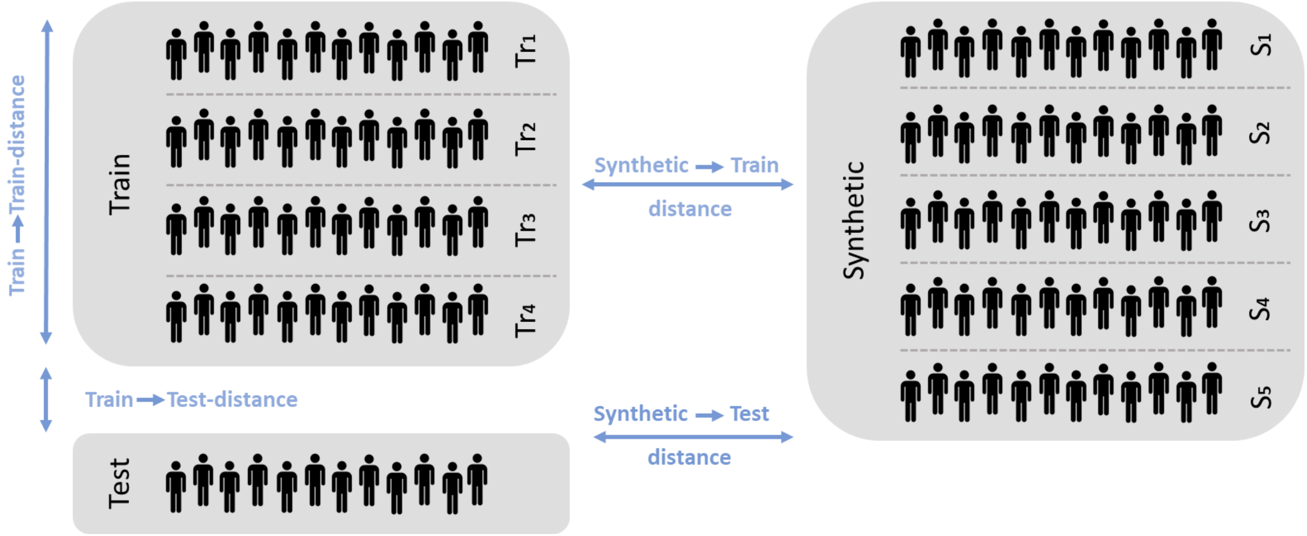

Supplementary Figure 2 Partitioning of privacy assessment subsets. Because of the mismatch between training set size (80% of the total cohort) and test set size (20%), both the training set and the synthetic cohort were partitioned into equally sized (20% each) subsets in order to guarantee adequate comparability via Hamming distance calculation.
